# Mortality in GOLD stages of COPD and its dependence on symptoms of chronic bronchitis

**DOI:** 10.1186/1465-9921-6-98

**Published:** 2005-08-25

**Authors:** Marie Ekberg-Aronsson, Kerstin Pehrsson, Jan-Åke Nilsson, Peter M Nilsson, Claes-Göran Löfdahl

**Affiliations:** 1 Department of Respiratory Medicine and Allergology, University of Lund, S-221 85 Lund, Sweden; 2 Department of Medicine, University of Lund, University hospital, S-205 02 Malmö, Sweden

**Keywords:** chronic bronchitis, COPD, epidemiology, FEV_1_, GOLD, mortality, respiratory symptoms, smoking

## Abstract

**Background:**

The GOLD classification of COPD severity introduces a stage 0 (at risk) comprising individuals with productive cough and normal lung function. The aims of this study were to investigate total mortality risks in GOLD stages 0–4 with special focus on stage 0, and furthermore to assess the influence of symptoms of chronic bronchitis on mortality risks in GOLD stages 1–4.

**Method:**

Between 1974 and 1992, a total of 22 044 middle-aged individuals participated in a health screening, which included a spirometry as well as recording of respiratory symptoms and smoking habits. Individuals with comorbidity at baseline (diabetes, stroke, cancer, angina pectoris, or heart infarction) were excluded from the analyses. Hazard ratios (HR 95% CI) of total mortality were analyzed in GOLD stages 0–4 with individuals with normal lung function and without symptoms of chronic bronchitis as a reference group. HR:s in smoking individuals with symptoms of chronic bronchitis within the stages 1–4 were calculated with individuals with the same GOLD stage but without symptoms of chronic bronchitis as reference.

**Results:**

The number of deaths was 3674 for men and 832 for women based on 352 324 and 150 050 person-years respectively. The proportion of smokers among men was 50% and among women 40%. Self reported comorbidity was present in 4.6% of the men and 6.6% of the women. Among smoking men, Stage 0 was associated with an increased mortality risk, HR; 1.65 (1.32–2.08), of similar magnitude as in stage 2, HR; 1.41 (1.31–1.70). The hazard ratio in stage 0 was significantly higher than in stage 1 HR; 1.13 (0.98–1.29). Among male smokers with stage 1; HR: 2.04 (1.34–3.11), and among female smokers with stage 2 disease; HR: 3.16 (1.38–7.23), increased HR:s were found in individuals with symptoms of chronic bronchitis as compared to those without symptoms of chronic bronchitis.

**Conclusion:**

Symptoms fulfilling the definition of chronic bronchitis were associated with an increased mortality risk among male smokers with normal pulmonary function (stage 0) and also with an increased risk of death among smoking individuals with mild to moderate COPD (stage 1 and 2).

## Background

Chronic obstructive pulmonary disease (COPD) is a major cause of increased morbidity and mortality [1]. The Global Initiative for Chronic Obstructive Lung Diseases (GOLD) guidelines were published in 2001 [2], and revised in 2004 [3] with the aim of increasing awareness of COPD and of decreasing morbidity and mortality from the disease. The GOLD staging system for COPD introduces a stage 0 (at risk), defined as the presence of chronic respiratory symptoms such as productive cough in individuals with preserved normal pulmonary function. The importance of the "at risk" stage, especially the effect of productive cough on morbidity and mortality, is an issue under debate [4]. To assess prognosis in patients with established COPD, the BODE-index for prediction of mortality (based on degree of airflow limitation, dyspnea, 6 min walking distance and body mass index) has been developed [5]. The BODE index is probably less helpful in the early stages of COPD when individuals may have no symptoms or symptoms of chronic bronchitis only ("smoker's cough"), regarded by most individuals (and physicians) as innocent symptoms. Contrary to this view, some previous studies have shown an increased mortality risk associated with symptoms of chronic bronchitis [6,7].

We hypothesized that chronic productive cough (symptoms of chronic bronchitis) could predict an increased mortality risk. The aims of this study were firstly to investigate all-cause mortality risks in relation to GOLD stages 0–4 with special focus on stage 0. Secondly, the effect of symptoms of chronic bronchitis on mortality risks in GOLD stages 1–4 was also assessed.

## Study Population and Methods

### Study population

**Figure 1:**
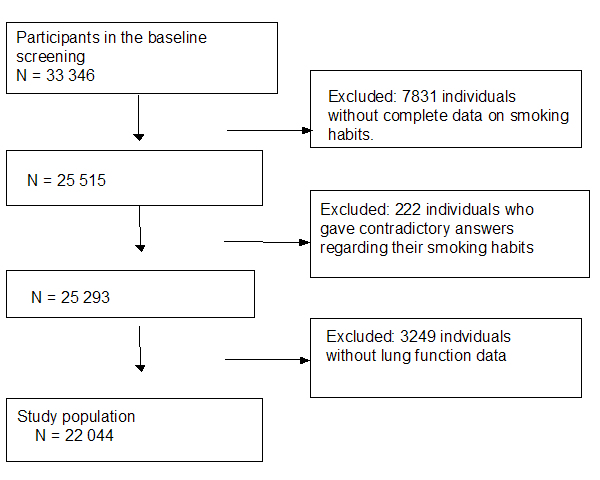
Flow chart of the study population.

With a population of 250000 inhabitants, Malmö is Sweden's third largest city. The Malmö Preventive Program (MPP), a preventive, case-finding programme for cardiovascular risk factors and alcohol abuse, was created in 1974 at the Department of Preventive Medicine of Malmö University Hospital. The aim of this programme was to screen large strata of the adult population, mostly middle-aged men and women born in prespecified years, in order to find high-risk individuals for preventive intervention [8,9]. Between 1974 and 1992, a total of 22,444 men (mean age 44 years range 27 – 61) and 10 902 women (mean age 50 years, range 28 – 58) attended the programme, with an overall attendance rate of 70% (range 64–78) [8]. The present study was based on prospectively collected data including detailed data on smoking habits obtained from 22,044 individuals with complete data on smoking habits and lung function among the individuals who took part in the MPP. The study population is described in fig 1.

### Screening procedure and intervention

All individuals who participated in the MPP took part in a comprehensive health screening, which included a physical examination, spirometry and blood tests. Additionally, lifestyle-related risk factors were assessed by means of a self-administered questionnaire including questions on smoking habits. Various interventions (lifestyle modification, drug therapy) were offered to nearly 25% of the screened individuals for shorter or longer periods [8,10]. Intervention against smoking was however only instituted if another cardiovascular risk factor was present and consisted of advice given by a nurse to stop smoking, sometimes supported by measurements of carboxyhemoglobin (COHb) and feedback information to the individual with a history of smoking [11].

### Recording of tobacco smoking

All individuals completed a self-administered questionnaire regarding their tobacco consumption and inhalation habits. Tobacco consumption was calculated by equating one cigarette to 1 gram, one cheroot to 3 grams, and one cigar to 5 grams of tobacco. Individuals were categorized according to their self-reported smoking habits. Individuals who gave negative answers to all smoking-related questions regarding current and previous smoking habits were classified as never smokers. Individuals who stated that they had stopped smoking and who gave negative answers to all other smoking-related questions were classified as former smokers. Individuals who stated that they were currently smoking were classified as smokers. Those who gave contradictory answers were excluded. In order to adjust for differences in the amount of tobacco smoked a categorical variable on tobacco consumption was used separating individuals who smoked 20 grams or more tobacco per day from those who smoked less than that amount.

### Assessment of pulmonary function

Pulmonary function was assessed by a screening spirometry. A Spirotron^® ^apparatus (Drägerwerk AG, Lűbeck, Germany) was used with the individual in an upright standing position without a nose clip. Specially trained nurses performed the tests. Forced expiratory volume in one second (FEV_1_) and forced vital capacity (FVC) were recorded. One acceptable manoeuvre with respect to the individual's cooperation and performance was required [12]. FEV_1 _was analysed as percent of predicted (pred) values. Predicted values of FEV_1 _were obtained from an internally derived linear regression based on height and age in a subgroup of 3467 male and 2961 female never-smokers.

Men: Pred. FEV_1 _(L): 4.422 × height (m) - 0.0381 × age (year) - 2.483, SD 0.63

Women: Pred. FEV_1 _(L): 3.615 × height (m) - 0.0217 × age - 2.134, SD 0.45

### Assessment of respiratory symptoms

Respiratory symptoms were assessed by a self-administered questionnaire. We used the classical definition of chronic bronchitis. The questions regarding chronic productive cough varied somewhat during the screening period. In the period from screening start on the 1st September 1974 until the 1st March 1978 the following question was used:

1."During any period of your life, have you had daily cough lasting more than three months in more than two years?" ("Ever symptoms of symptoms of chronic bronchitis")

In the period from 2nd of March 1978 until the end of the screening the following two questions were used *:*

2. "During the last two years have you had daily cough lasting for more than 3 months", and

3. "During the last two years, have you had phlegm coming up from your chest daily for more than three months? ("Recent symptoms of chronic bronchitis")

Individuals who answered affirmatively to the first question ("ever symptoms of chronic bronchitis" or both the second and third question ("recent symptoms of chronic bronchitis") were regarded as having symptoms of chronic bronchitis corresponding to the definition of GOLD stage 0.

### Classification of pulmonary function

GOLD Stages 0–4 were defined as follows:

stage 0 FEV_1_/FVC ≥ 0.70 and FEV_1 _≥ 80% pred and symptoms of chronic bronchitis,

stage 1 (mild): FEV_1_/FVC < 0.70 and FEV_1 _≥ 80% pred,

stage 2 (moderate): FEV_1_/FVC < 0.70 and FEV_1 _< 80% pred and FEV_1 _≥ 50% pred,

stage 3 (severe): FEV_1_/FVC < 0.70 and FEV_1 _< 50% pred and FEV_1 _≥ 30% pred,

stage 4 (very severe) : FEV_1_/FVC < 0.70 and FEV_1 _< 30% pred,

Individuals without symptoms of chronic bronchitis and with FEV/FVC ≥ 70 and FEV_1 _≥ 80% predicted were classified as normal and used as a reference group throughout the study except for the analyses of the effect of symptoms of chronic bronchitis within stage 1–3 where individuals with the same GOLD stage, but without symptoms of chronic bronchitis were used as reference.

### Assessment of comorbidity

Baseline comorbidity was assessed by a self-administered questionnaire. Individuals who answered affirmatively to any of the following four questions were regarded as having significant comorbidity and were excluded from further analyses.

1. "Have you previously had a stroke?"

2. "Have you a physician's diagnosis of angina pectoris?"

3. "Do you have diabetes?"

4. "Have you been hospitalised due to a heart infarction?"

5. "Have you previously or currently received a diagnosis of cancer?"

### Register follow-up analyses

All individuals were followed up in national registers for total mortality until 31^st ^December 2003. Cause-specific mortality was assessed in male smokers of GOLD stage 0. Notably, data on cause-specific mortality was only available until 31^st ^December 2002. The Swedish Board of Health and Welfare provided data from national registers on death certificates and cancer diagnoses. The overall autopsy rate was 44% during the study period. The cases were coded according to the International Classification of Diseases ICD 8–10. Mortality for all causes of death, cardiovascular disease (ICD 8–9: 401–414, 424, 426–429, 431–444; ICD 10: I10–25, I34–37, I44–49, I50–52, I61–71), all cancer except for lung cancer (ICD 8–9: 140–208; ICD 10: C00–C97), lung cancer (ICD 8–9: 162–165; ICD10: C34, 38, 39, 45) and respiratory diseases (ICD 8–9: 460–466, 480–487, 490–496, 500–503, 510–519; ICD10: J00–99) were recorded. The vital status at December 31^st ^2003 was unknown in 412 individuals who had left the country by that time. The accumulated person-years until they left the country were used in the analyses.

### Data analysis

The computer-based analysis programme, Statistical Programme for Social Sciences (SPSS) version 10.0, was used for all calculations. The Cox Proportional Hazards Regression Model [13] was used to calculate relative total mortality rates in GOLD stages 0–4 with adjustments for age only in never-smokers and former smokers. Cause specific mortality was analysed only in smoking men of stage 0. In smokers, adjustments were also made for tobacco consumption (<20 grams/≥20 grams per day) and inhalation habits (yes/no). The reference group in all analyses consisted of individuals with normal pulmonary function and without symptoms of chronic bronchitis. In order to address possible differences in mortality risk between "ever symptoms of chronic bronchitis and "recent symptoms of chronic bronchitis" we calculated the mortality risk in GOLD stage 0 among smoking men using both "ever symptoms of chronic bronchitis" and "recent symptoms of chronic bronchitis" for the definition of stage 0, with the same adjustments and reference group as described above. Among smokers, the influence of symptoms of chronic bronchitis was assessed comparing individuals with and without symptoms of chronic bronchitis within the GOLD stages 1–4 after adjustment for age, tobacco consumption and inhalation habits. All variables were included in the analyses as categorical (yes/no) except for age. All individuals with self-reported comorbidity at baseline were excluded from the analyses. All analyses were performed separately for men and women. A p-value of less than 0.05 was considered statistically significant.

## Results

### Characteristics of the study population

The total number of deaths was 3674 among men and 832 in women based on 352 324 and 150 050 person-years, respectively. Among men of GOLD stage 0, 217 individuals had "ever symptoms of chronic bronchitis" and 101 individuals had "recent symptoms of chronic bronchitis". The corresponding figures among women were 33 and 142.

The proportion of current smokers was high at the time of screening and slightly higher among men (50%) than among women (40%). Former smokers were also more common among men (27%) than among women (16%). Among men, 24% of the individuals and among women, 40% stated that they had never smoked (never smokers). Self-reported comorbidity was present in 4.6% of the men and 6.6% of the women. The prevalence of COPD in the study population was for men in GOLD stage 0–4: 2.2%, 9.9%, 6.9%, 1.0%, and 0.3%. The corresponding figures among women were: 2.4%, 4.5%, 4.3%, 0.6%, and 0.2% respectively, (Table 1).

**Table 1:** Characteristics of the study population*

	**Men**	**Women**
Number (n)	14 630	7414
Smoker	7221 (49.4)	2961 (39.9)
Exsmoker	3942 (26.9)	1169 (15.8)
Never-smoker	3467 (23.7)	2961 (39.9)
Age	46.4 (5.7)	47.5 (7.8)
Follow-up time	22.2 (5.7)	20.2 (4.7)
Person-years	352 324	150 050
Deaths	3674 (25.1)	832 (11.2)
Reference**	10215 (69.8)	5706 (77)
Not classif***	1453 (9.9)	821 (11.0)
Gold 0	318 (2.2)	175 (2.4)
Gold 1	1443 (9.9)	336 (4.5)
Gold 2	1010 (6.9)	316 (4.3)
Gold 3	149 (1.0)	48 (0.6)
Gold 4	42 (0.3)	12 (0.2)
Comorbidity****	676 (4.6)	489 (6.6)

* Data are given as numbers (%), but age and follow-up time are given as years (mean, standard deviation). ** Normal pulmonary function, without symptoms of chronic bronchitis

*** Not-classif: individuals not classified as normal or categorized according to the GOLD stages.

**** Self-reported previous/current stroke, angina pectoris diagnosed by a physician, diabetes, hospitalization due to a heart infarction or a diagnosis of cancer at baseline.

### Mortality risks in smokers

Among smoking men the hazard ratio (HR) increased stepwise from stage 1 to stage 4 (p for trend < 0.0001). Stage 1 showed a slightly increased HR (1.13) of borderline significance and the HR increased further in stage 2 (1.41). In the later stages a pronounced increase in mortality risk was seen. In stages 3 and 4 the risks increased two- to tenfold, respectively, suggesting a possible threshold with respect to an increased mortality risk at a FEV1 equal to or lower than 50% predicted. Among smoking men, stage 0 was associated with significantly elevated mortality risk, HR: 1.65 (1.32–2.08), of similar magnitude as in stage 2, HR: 1.41 (1.31–1.70), suggesting an adverse influence of symptoms of chronic bronchitis on mortality risks in these symptomatic smokers. The relative risk in stage 0 was significantly higher than in stage 1; HR: 1.13 (0.98–1.29). No difference in mortality risk was seen among smoking men of GOLD stage 0 when we used "ever symptoms of chronic bronchitis" (217 individuals), HR: 1.62 (1.25–2.12 p-value: <0.0001) or "recent symptoms of chronic bronchitis" (101 individuals) HR: 1.74 (1.13–2.68 p-value: 0.012) to define stage 0. Among smoking women, as for men, the HR:s in stage 1–4 increased stepwise (p for trend <0.0001). There was an overlap in the confidence intervals in stages 1 and 2 with no significant difference between the stages. As for men, GOLD 3 and 4 conveyed an increase in mortality risk that was significantly higher than in the other GOLD stages, again suggesting a possible "threshold" at a FEV_1 _of 50% predicted. The HR was slightly increased in stage 0, with a similar magnitude as in smoking men but of borderline significance, probably due to low statistical power or differences in the influence of symptoms of chronic bronchitis between genders, (Table 2).

**Table 2:** Mortality risks in GOLD stages 0–4 stratified for smoking status and gender*

	Smoker***					Former smoker***					Never smoker***				
**Men**	N*	Deaths	RR**	CI	P-value	N*	Deaths	RR	CI	p-value	N*	Deaths	RR	CI	p-value

Ref	4430	1205				2993	517				2792	404			
Gold 0	215	92	1.65	1.32–2.08	<0.0001	54	17	1.75	1.05–2.43	0.033	49	6	1.03	0.46–2.31	0.95
Gold 1	792	269	1.13	0.98–1.29	0.087	405	68	0.80	0.61–1.05	0.10	246	51	1.31	0.97–1.76	0.080
Gold 2	710	309	1.41	1.31–1.70	<0.0001	173	44	1.16	0.81–1.66	0.43	127	32	1.73	1.18–2.54	0.005
Gold 3	101	63	2.42	1.84–3.18	<0.0001	33	18	2.42	1.44–4.08	0.001	15	7	3.93	1.86–8.30	<0.0001
Gold 4	27	21	3.57	2.23–5.71	<0.0001	8	4	2.59	0.83–8.07	0.10	7	2	1.04	0.15–7.39	0.97
**Women**															
Ref	2174	256	1			993	87	1			2539	168	1		
Gold 0	108	17	1.64	0.98–2.73	0.060	25	1	0.53	0.07–3.79	0.53	42	7	3.12	1.46–6.67	0.003
Gold 1	184	39	1.75	1.22–2.51	0.002	46	2	0.23	0.03–1.65	0.14	106	8	0.83	0.37–1.87	0.64
Gold 2	234	52	1.75	1.27–2.42	0.001	32	5	1.48	0.59–3.67	0.40	50	7	2.11	0.99–4.52	0.054
Gold 3	36	19	5.11	3.09–8.45	<0.0001	5	1	NA			7	2	3.91	0.96–15.8	0.056
Gold 4	9	6	10.26	4.53–23.25	<0.0001	0	0	NA			3	1	18.10	2.53–129.6	0.0004

* Individuals with self-reported baseline comorbidity (current/previous cancer, stroke, angina pectoris, heart infarction or diabetes) were excluded. GOLD stage 4 were not analysed due to low number of cases. The reference group consists of individuals without chronic bronchitis and with normal pulmonary function.

** HR denotes hazard ratio with CI 95% confidence interval.

*** In smokers adjustments were made for age, inhalation habits (yes/no) and tobacco consumption (>=20 g/<20 g tobacco). In former and never-smokers adjustments were made for age only.

### Cause specific mortality among male smokers with GOLD stage 0

Among male smokers HR for cardiovascular mortality was (16 deaths): 0.88 (0.53–1.44), p-value 0.60, mortality in all cancer mortality except lung cancer: (22 deaths): 2.03 (1.31–3.15) p-value 0.001, mortality in lung cancer: (12 deaths) 2.22 (1.23–4.02), p-value: 0.008, other causes: (24 deaths) 2.05 (1.45–2.88) p-value: <0.0001. Respiratory mortality was not analysed due to a lower number of deaths, (only one death in respiratory diseases was recorded in stage 0).

### Mortality risks among former smokers and never smokers

Among male former smokers the numbers of deaths were generally low except for stages 1 and 2, which showed no increased mortality risk. Stage 0 was associated with an increased risk, however the number of deaths was low and the results should therefore be interpreted with caution. This was also the case among male never smokers except for stage 2 (32 deaths) that showed a significantly increased HR of 1.73 (1.18–2.54), suggesting an effect of low FEV_1 _also among never-smokers. Among former and never-smoking women, the numbers of deaths were very low in all GOLD stages and the results should therefore be interpreted with caution, (Table 2).

### The influence of symptoms of chronic bronchitis on mortality risks in stages 1 and 2

We compared subjects with and without self-reported symptoms of chronic bronchitis at baseline and found that the HR was significantly increased among male smokers with symptoms of chronic bronchitis and mild COPD (stage 1, with airflow limitation only) as compared to those without symptoms of chronic bronchitis. Among smoking women this was also the case among individuals with moderate COPD (stage 2) suggesting an effect of symptoms of chronic bronchitis on mortality risks in the early stages of COPD, (Table 3).

**Table 3:** Mortality risks among smokers within GOLD stages 1–4 in individuals with symptoms of chronic bronchitis, with individuals from the same GOLD stage without symptoms of chronic bronchitis as reference*

**Gold stage**	**Chr Br (%)**	**Deaths**	HR**	**95%CI**	**p-value**
**Men**					
GOLD 1	44 (5.8)	25	2.04	1.34–3.11	0.001
GOLD 2	55 (8.3)	26	1.20	0.80–1.80	0.38
GOLD 3	17 (18.7)	9	0.71	0.34–1.51	0.38
GOLD 4	5 (20.8)	2	0.41	0.09–1.87	0.25
					
**Women**					
GOLD 1	4 (2.4)	1	1.58	0.22–11.79	0.66
GOLD 2	17 (8.1)	7	3.16	1.38–7.23	0.006
GOLD 3	7 (21.2)	3	2.19	0.27–18.02	0.47
GOLD 4	3 (37.5)	2	0.58	0.08–4.11	0.59

* HR denotes hazard ratio, with CI: 95% confidence interval, Chr Br: symptoms of chronic bronchitis.

**Adjusted for age, tobacco consumption (>=20 g/<20 g tobacco) and inhalation habits.

## Discussion

We investigated mortality risks in relation to the GOLD classification of obstructive pulmonary disease in a large population-based study of middle-aged Swedish men and women. The main findings were firstly that among smokers, symptoms of chronic bronchitis were associated with increased total mortality risk among individuals with normal pulmonary function (corresponding to GOLD stage 0) and among those with mild to moderate COPD (GOLD stage 1 and 2). Secondly, among male smokers of stage 0, cause-specific mortality showed increased HR for all cancer (except lung cancer), lung cancer and other diseases. Thirdly, the total mortality hazard ratio in stages 1–4 increased stepwise with a pronounced increase in risk at a level of FEV1 below 50% predicted, suggesting a threshold effect in mortality risk at this level. Individuals with comorbidity of cardiovascular disease, diabetes and cancer at baseline were excluded and we have adjusted for age and smoking habits as completely as possible including tobacco consumption and inhalation habits.

### Comparison with other studies

#### Prevalence of stage 0

The prevalence of individuals with stage 0 in our study was 2.2% in men and 2.4% in women. In the Copenhagen City Heart study the prevalence was slightly higher; criteria for GOLD Stage 0 were met in 5.8% of the total adult population and in 7.2% of smokers [4]. In the National Health and Nutrition Examination Study (NHANES) study [14] the prevalence of respiratory symptoms among individuals with normal pulmonary function was substantially higher (approximately 13%). This is probably mostly dependent on different selection criteria due to variation in the definition of stage 0. Vestbo and coworkers limited the selection of individuals to those with symptoms of chronic bronchitis but included all individuals without airflow limitation irrespective of the level of FEV_1_. On the other hand the NHANES study had higher prevalence rates probably due to a broader definition of the respiratory symptoms [14]. In our study a specific definition of symptoms of chronic bronchitis was used and we only selected individuals with normal pulmonary function, both with respect to FEV_1 _and the FEV_1_/FVC ratio. Consequently the prevalence of stage 0 was lower in our study than in the NHANES [14] and the Copenhagen City Heart study [4].

#### Mortality risks associated with symptoms of chronic bronchitis

Among male smokers of our study, the HR of total mortality in GOLD stage 0 was strongly significant irrespective of whether we used "ever" or "recent" symptoms of chronic bronchitis" to define stage 0. The HR:s were of a similar magnitude as for stage 2. Among men with GOLD stage 1 in men and among women with stage 2 in women, increased HR:s were noticed in smokers who reported symptoms of chronic bronchitis, implying that respiratory symptoms might be important for an adverse prognosis. However, in GOLD stage 3 we were not able to show an additive risk associated with symptoms of chronic bronchitis possibly due to low statistical power.

Similar results have previously been demonstrated in some studies. In the Copenhagen City Heart study, chronic mucus hypersecretion was associated with an increased risk of all cause mortality statistically significant in men only (relative risk: 1.3 in men and 1.1 in women) [7]. There was no interaction between the effect of chronic mucus hypersecretion and FEV_1 _percent predicted with regard to total mortality which is in agreement with our results. However, also in the same study, regarding obstructive lung disease mortality, the effect of chronic mucus hyper-secretion varied with the level of ventilatory function, being weak in individuals with normal ventilatory function but more pronounced in individuals with reduced ventilatory function. This interaction was statistically significant [7]. Furthermore, another study using the Copenhagen City Heart study population showed that chronic mucus hyper-secretion was associated with later hospitalization due to COPD, relative risks were 2.4 (1.3 to 4.5) for men and 2.6 (1.2 to 5.3) for women [16].

In contrast, in the National Health and Nutrition Examination Study (NHANES) the presence of respiratory symptoms was not associated with an increased mortality risk in subjects with normal lung function [14]. The relative risk (1.2) was elevated but not statistically significant probably due to the fact that a much wider definition of respiratory symptoms was used which included as well individuals with wheeze and a diagnosis of asthma. In our study based on a similar number of deaths, but also on a more specific definition of respiratory symptoms (symptoms of chronic bronchitis) a HR of 1.6 was statistically significant among smoking men.

Occupational studies have shown results in agreement with our study. In a population of 1061 men working in the Paris area and followed for 22 years, chronic phlegm production was significantly associated with mortality: Relative risk 1.35; (p less than 0.01) [6]. In a group of gold miners, chronic mucus hyper-secretion was not related to COPD mortality, but related to mortality in ischemic heart disease and other causes after adjustment for tobacco and dust exposure [17].

On the contrary, in another occupational cohort of 2,718 British men examined between 1954 and 1961, and followed for 20 to 25 years, the risk of death from COPD was correlated with the initial degree of air-flow obstruction. Among men with similar initial air-flow obstruction, age-specific COPD death rates were not significantly related to initial mucus hypersecretion [18].

The explanation for the relation between chronic productive cough and future mortality risk is still unclear. Previously, in the classic study by Fletcher and coworkers, conducted in a cohort of working men in London, chronic phlegm production was unrelated to the development of airway obstruction and regarded as a less important condition [19]. Furthermore, the presence of stage 0 at baseline was not a meaningful predictor for the subsequent development of COPD in later stages after 5 and 15 years follow-up in a Danish prospective population based study [4].

Thus it seems that the symptoms of chronic bronchitis do not affect the FEV_1 _decline and development of COPD to the same extent that it affects all-cause and obstructive lung disease mortality and risk of COPD hospitalization. An explanation could be that symptoms of chronic bronchitis may be an independent pulmonary disorder associated with inflammatory and/or infectious exacerbations [20] and possibly also with the development of other diseases such as lung cancer [15] and thus not specifically associated with COPD development and progression. We were able to analyse cause specific mortality in GOLD stage 0 in male smokers. Our findings of significantly increased HR for lung cancer, all cancers, and other diseases in individuals of stage 0 support this view.

#### Mortality risks in stages 1–4

GOLD stage 1 in our study, as well as in the NHANES study [14], conveyed only a slight increase in total mortality risk of borderline significance, even lower than found for GOLD stage 0. Among former smokers there was a tendency towards a decreased risk. Thus among individuals who quit smoking when still in GOLD stage 1, the future mortality risk will be normal. In GOLD stages 2 to 4 our findings of increased mortality HR are in agreement with other previously conducted studies, as they actually reflect a successive reduction in FEV_1 _[21-23]. Mortality risks were also increased among never smokers, and these results are entirely in line with other studies [24]. The NHANES study showed increased mortality risks associated with moderate to severe COPD, among smokers and former smokers of approximately the same magnitude as we report [14].

### Study limitations

A methodological limitation of these findings is the measurement of pulmonary function. The protocol of the lung function tests did not meet the standards of current recommendations [12]. The values of the internal regression equation based on height and age and the standard deviation of the equation is similar to that of the Working party of the European Community for Steel and Coal [25]. Hence, we believe that the methodological error is similar to the one achieved after optimal spirometric technique. However there remains the possibility of misclassification due to decreased specificity in the spirometries which could have resulted in an underestimation of the results rather than the reverse.

Another possible limitation of this study is dependent on the fact that smoking prevalence in Sweden has decreased since the 1970's [26]. Some smokers in this study are likely to have stopped smoking during the follow-up time. Smoking habits were assessed at one point in time and not repeated thereafter. Hence, we have not been able to calculate accumulated smoking exposure in terms of individual pack-years. However, even the use of pack-years has its limitations, especially with respect to recall biases in subjects with a long history of smoking [27].

Furthermore, we have no follow-up data on symptoms of chronic bronchitis and thus we were not able to determine whether the symptoms of chronic bronchitis were stable or not. According to previous studies symptoms of chronic bronchitis may disappear in some individuals [4]. In those individuals, symptoms of chronic bronchitis may have a limited influence on mortality risks and could cause an underestimation of the effect of persistent symptoms of chronic bronchitis in this study. In addition, we have no spirometric follow-up data and hence we are not able to determine whether individuals with GOLD stage 0 at baseline have developed COPD in the later GOLD stages during the follow up time.

Another limitation of the study was the lower statistical power in the analyses of women. Our findings in women, especially in former smokers and never smokers should therefore be interpreted with caution.

### Implications of our findings

We believe that symptoms of chronic bronchitis should be regarded as a marker for an increased all-cause mortality risk. Our data show that among middle-aged smokers, symptoms of chronic bronchitis might represent an independent risk factor for an early mortality equal to that of having moderate COPD.

It is however uncertain from previous studies whether symptoms of chronic bronchitis really are related to the development and progression of COPD [4]. An increased risk of COPD mortality associated with chronic mucus hyper secretion among individuals with established COPD has been shown previously [7]. In our study, symptoms of chronic bronchitis increased the risk of all cause mortality and mortality in cancer, lung cancer and other causes in smoking individuals with normal lung function (stage 0) and the risk of total mortality in individuals with GOLD stage 1 and 2. Our findings indicate that symptoms of chronic bronchitis might be an independent pulmonary disorder that may or may not be associated with COPD. It is therefore questionable whether GOLD stage 0 should be used as an "at risk" stage for COPD development in future COPD classifications.

Finally, GOLD stage 0 indicates a group of individuals with a very unfavourable prognosis similar to that of individuals with GOLD stage 2. Among smokers, symptoms of chronic bronchitis contributed to an adverse prognosis not only in individuals "at risk", but also in those with early stages of COPD with GOLD 1 and 2.

## Competing interests

The author(s) declare that they have no competing interests.

## Authors' contributions

Marie Ekberg-Aronsson: Taking part in the conception and design of the study. Performed the statistical analyses and interpretation of data, drafting and revising the article, giving final approval to the version to be published. 

Kerstin Pehrsson: Taking part in the conception and design of the study. Interpretation of data and revising the article, giving final approval to the version to be published.

Jan-Åke Nilsson: Taking part in the conception and design of the study. Statistical analysis and interpretation of data, giving final approval to the version to be published.

Peter Nilsson: Taking part in the conception and design of the study, acquisition and interpretation of data and revising the article, giving final approval to the version to be published.

Claes-Göran Löfdahl: Taking part in the conception and design of the study, interpretation of data and revising the article, giving final approval to the version to be published.
